# Supplementary material for: Preparedness of tertiary care hospitals to implement the national TB infection prevention and control guidelines in Bangladesh: A qualitative exploration
Source: PLoS One. 2022 Feb 3;17(2):e0263115. doi: 10.1371/journal.pone.0263115 (PMC8812944; doi:10.1371/journal.pone.0263115)
Supplement: S1 Appendix — (DOCX) [file pone.0263115.s001.docx]

**S1 Appendix: Guidelines for key informant interviews**

- *Age, sex, marital status, education, religion, occupation.*
- *What are the current activities of GOB National TB Control Programme being implemented at this hospital?*
- *Is there any coordinating body or responsible person for TB infection control in place?*
- *Does the facility have any written infection control plan in place?*
- *Does this hospital have the capacity to*
- *Managerial*
  - *Identify and strengthen a coordinating body for infection control*
  - *Develop a comprehensive national infection control strategy, guidelines and policies*
  - *Conduct a situational analysis of the current risk level and implementation of TB-IC to contribute to the development of the national infection control strategy*
  - *To design, construction, renovation, use and maintenance of TB wards*
  - *Engage decision-makers and key stakeholders in TB IC advocacy, communication and social mobilization.*
  - *TB surveillance and assessment of TB among health care workers*
  - *Include TB/airborne IC in the formulation of policies and work plans*
  - *Monitoring and evaluation of infection control healthcare measures*
- *Administrative*
  - *Rapid identification of people with TB symptoms*
  - *Separate infectious patient*
  - *Control the spread of pathogens*
  - *Minimize time spent in health facilities*
  - *Educate staff on signs and symptoms of TB and encourage early care seeking*
- *Environmental and*
  - *Type of ventilation system? How can we make the best use of the ventilation system?*
  - *Purchase equipment to measure air change per hour in the in-patient ward*
  - *Conduct (periodic) air exchange measurements and identify the areas in the facility with insufficient air exchange*
  - *Use of upper room UVGI fixtures*
  - *Establishing appropriate bio-safety measures*
- *Personal protective equipment*
  - *Does the facility have available supply of N95 mask for staff?*
  - *Did the facility arrange fit testing and/or fit check for N95 masks?*
  - *Provide information to patients and staff why staff is wearing respirators and patients wear face masks*
- *Staff training in TB infection control*
- *How can the national TB IC policy be made most context appropriate?*
