# Supplementary material for: Preparedness of tertiary care hospitals to implement the national TB infection prevention and control guidelines in Bangladesh: A qualitative exploration
Source: PLoS One. 2022 Feb 3;17(2):e0263115. doi: 10.1371/journal.pone.0263115 (PMC8812944; doi:10.1371/journal.pone.0263115)
Supplement: S2 Appendix — (DOCX) [file pone.0263115.s002.docx]

**S2 Appendix. Focus group discussion guideline**

- *Age, sex, education, religion, employment history-duration, position.*
- *How often do you deal with TB patient?*
- *Do you think TB is a serious disease? If yes, why, if no, why?*
- *What do you do with infectious patients and how do you separate them? How much time it takes to diagnose a confirm TB patients? How can it be improved to improve rapid diagnosis of pulmonary TB patients?*
- *Are you involved in screening TB patient by any means (sputum collection, CXR, Gene Xpert)- please describe in details.*
- By which means do you consider a person as TB patient? Why?
- What do you think about screening TB patient by symptoms?
- *How many presumptive TB patients you attend/screen in a typical day?*
- *When a presumptive TB patient comes to your facility, what steps do you take? What capacity the facility has to follow the steps? Barriers and facilitators to follow the steps?*
- *What steps do you take to prevent the contamination of air by mycobacterium tuberculosis exhaled by pulmonary TB patients? What capacity the facility has to follow the steps? Barriers and facilitators to follow the steps?*
- *What protective health care measures are available in your facility? Did you use them? If yes, how often? If no, why?*
- *When a TB patient is admitted, what steps do you take to protect yourself?*
- *Did you use any facial protection (cloth masks, surgical mask and 95 respirators)? How would you compare N95 respirators with the cloth/surgical mask?*
- *What steps do you take for TB patient management? (cough etiquette, isolation and cohorting, bed distribution)? What capacity the facility has to follow these steps? Barriers and facilitators to follow these steps?*
- *In your opinion, what are the positive consequences of wearing a N95 respirators?*
- *What difficulties do you have while wearing the N95 respirators? (For probe: Hot? Heard to breathe through, difficulties to communicate with patients, patient/other caregivers discouraged you wearing mask?)*
- *What factors motivate you to wear a N95 respirator?*
- *Do you feel any discomfort during wearing mask? If yes, what type of discomfort?*
- *Do you think N95 respirator can protect you from being infected? Do you feel protective during wearing a N95 respirator?*
- *What factors remind you to wear a N95 respirator?*
- *What are the critical times for wearing a N95 respirator and why? How long should we wear a respirator? And why?*
- *After using, how would you store the 95 respirators for further use?*
- *After using, how would you store the surgical masks for further use?*
- *After using, how would you store the cloth masks for further use?*
- *What are the sources of 95 respirators, cloth masks and surgical masks?*
- *In your opinion, what are the quality of these respirator, cloth masks and surgical masks?*
- *What are the difficulties you face to prompt identification of people with TB symptoms?*
- *What initiative do you take to control the spread of pathogens? What are advantages and disadvantages you face while implementing this activity?*
- *How long a TB patient stay in your facility and why? What initiatives can be taken to minimize time spent by pulmonary TB patients in health facilities*
