# Supplementary material for: Preparedness of tertiary care hospitals to implement the national TB infection prevention and control guidelines in Bangladesh: A qualitative exploration
Source: PLoS One. 2022 Feb 3;17(2):e0263115. doi: 10.1371/journal.pone.0263115 (PMC8812944; doi:10.1371/journal.pone.0263115)
Supplement: S1 Data — (DOCX) [file pone.0263115.s003.docx]

| **Designation** | **Profession** | **Age** | **Sex** | **Year of experience** | **Working ward** |
| --- | --- | --- | --- | --- | --- |
|  | Doctor | 54 | Male | 28 | Hospital |
|  | Doctor | 48 | Male | 22 | Unit Head, M-3 |
|  | Doctor | 49 | Male | 23 | Unit Head, M-1 |
|  | Doctor | 52 | Male | 26 | Unit Head, Peadiatric |
|  | Doctor | 59 | Male | 34 | Unit Head, Peadiatric |
|  | Doctor | 46 | Female | 22 | Peadiatric Unit-1 |
|  | Doctor | 52 | Male | 26 | Radiology |
|  | Doctor | 53 | Male | 27 | Pathology |
|  | Nurse | 54 | Female | 32 | Hospital |
|  | Nurse | 58 | Female | 35 | Hospital |
|  | Nurse | 35 | Male | 12 | Medicine Unit-4 |
|  | Doctor | 32 | Male | 6 | Emergency |
|  | Doctor | 32 | Male | 6 | Medicine Unit-2 |
|  | Doctor | 28 | Male | 2 | Medicine Unit-1 |
|  | Administrative staff | 34 | Male | 6 | Hospital |
|  | Administrative staff | 47 | Male | 24 | Hospital |

**Demographic information of Key Informants**

**Demographic information of FGD participants of RMCH**

| **Designation** | **Profession** | **Age** | **Sex** | **Year of Experience** | **Working Ward** |
| --- | --- | --- | --- | --- | --- |
|  | Doctor | 31 | Male | 2 | Ward-14 |
|  | Doctor | 32 | Male | 3.5 | Ward-15 |
|  | Doctor | 33 | Male | 3 | Ward-15 |
|  | Doctor | 34 | Male | 2.5 | Ward-15 |
|  | Doctor | 36 | Male | 10 | Ward-13 |
|  | Doctor | 32 | Female | 9 | Ward-13 |
|  | Doctor | 31 | Male | 5 | Ward-15 |
|  | Doctor | 34 | Male | 6 | Ward-37 |
|  | Doctor | 32 | Male | 3 | Ward-49 |
|  | Doctor | 30 | Male | 3 | Ward-49 |

**Demographic information of FGD participants of RMCH**

| **Designation** | **Profession** | **Age** | **Sex** | **Years of Experience** | **Working Ward** |
| --- | --- | --- | --- | --- | --- |
|  | Nurse | 50 | Female | 23 | Ward-14 |
|  | Nurse | 36 | Male | 6 | Ward-14 |
|  | Nurse | 48 | Female | 21 | Ward-14 |
|  | Nurse | 33 | Female | 3 | Ward-15 |
|  | Nurse | 50 | Female | 20 | Ward-15 |
|  | Nurse | 50 | Male | 21 | Ward-17 |
|  | Nurse | 34 | Female | 6 | Ward-13 |
|  | Nurse | 29 | Female | 3 | Ward-13 |
|  | Nurse | 24 | Female | 3 | Ward-13 |

**Demographic information of FGD participants of SBMCH**

| **Designation** | **Profession** | **Age** | **Sex** | **Years of experience** | **Working Ward** |
| --- | --- | --- | --- | --- | --- |
|  | Doctor | 34 | Male | 3.5 | MU-2 |
|  | Doctor | 32 | Male | 6 | MU-2 |
|  | Doctor | 36 | Male | 4 | MU-3 |
|  | Doctor | 37 | Male | 4 | MU-3 |
|  | Doctor | 36 | Male | 10 | MU-3 |
|  | Doctor | 33 | Male | 7 | MU-3 |
|  | Doctor | 41 | Male | 15 | SU-3 |
|  | Doctor | 28 | Female | 6M | MU-4 |

| **Designation** | **Profession** | **Age** | **Sex** | **Years of Experience** | **Working Ward** |
| --- | --- | --- | --- | --- | --- |
| SSN | Nurse | 46 | Female | 2 | MU-2 |
| SSN | Nurse | 51 | Female | 4 | MU-2 |
| SSN | Nurse | 48 | Female | 16 | FMU-1 |
| SSN | Nurse | 39 | Female | 4 | FMU-2 |
| SSN | Nurse | 27 | Female | 3 | FMU-3 |
| SSN | Nurse | 27 | Male | 1.5 | MU-4 |
| SSN | Nurse | 44 | Female | 3 | MU-1 |
| SSN | Nurse | 27 | Female | 10M | FMU-4 |
