# Supplementary material for: Preparedness of tertiary care hospitals to implement the national TB infection prevention and control guidelines in Bangladesh: A qualitative exploration
Source: PLoS One. 2022 Feb 3;17(2):e0263115. doi: 10.1371/journal.pone.0263115 (PMC8812944; doi:10.1371/journal.pone.0263115)
Supplement: Attachment — Submitted filename: PONE-D-21-24921 (1).pdf [file pone.0263115.s004.pdf]

PLOS ONE

Exploring hospital readiness for implementing the National TB infection control guidelines in two public tertiary care hospitals, Bangladesh

--Manuscript Draft--

**Manuscript Number:**	PONE-D-21-24921
**Article Type:**	Research Article
**Full Title:**	Exploring hospital readiness for implementing the National TB infection control guidelines in two public tertiary care hospitals, Bangladesh
**Short Title:**	Hospital readiness for implementing TB infection prevention and control guidelines
**Corresponding Author:**	Md Saiful Islam, MSS, MPH<br>International Centre for Diarrhoeal Disease Research Bangladesh<br>Dhaka, BANGLADESH
**Keywords:**	Pulmonary tuberculosis, patient management, nosocomial infection, Infection prevention and control, Bangladesh
**Abstract:**	In high tuberculosis (TB) burden countries, health settings, including non-designated TB hospitals, host many patients with pulmonary TB. Bangladesh's National TB Control Program (NTP) aims to strengthen TB infection prevention and control (IPC) in health settings. However, there has been a failure to date to assess the readiness of hospitals to comply with the recommendations. To address this gap, our study examined healthcare workers (HCW) knowledge and attitudes towards TB IPC guidelines and their perceptions regarding the hospitals' readiness, Bangladesh. Between January to December 2019, we conducted 16 key-informant interviews and four focus group discussions with HCW from two public tertiary care hospitals. In addition, we undertook a review of 13 documents [i.e., hospital policy, annual report, staff list, published manuscript]. Our findings showed that HCW acknowledged the TB risk and were willing to implement the TB IPC measures but identified key barriers impacting implementation. Gaps were identified in: policy (no TB policy or guidelines in the hospital), health systems (lack of TB IPC program, training and education, absence of healthcare-associated TB infection surveillance, low priority of TB IPC, no TB IPC monitoring and feedback, high patient load and bed occupancy, and limited supply of IPC resources) and behavioural factors (risk perception, compliance, and self-stigma). The additional service-level gap was the lack of electronic medical record systems. These findings highlighted that while there is a demand amongst HCW to implement TB IPC measures, the public tertiary care hospitals have got key issues to address. Therefore, the NTP may consider these gaps, provide TB IPC guidelines to these hospitals, assist them in developing hospital-level IPC manual, provide training, and coordinate with the ministry of health to allocate separate budget, staffing, and IPC resources to implement the control measures successfully.
**Order of Authors:**	Md Saiful Islam, MSS, MPH
	Sayeeda Tarannum
	Sayera Banu
	Kamal Ibne Amin Chowdhury
	Arifa Nazneen
	Abrar Ahmad Chughtai
	Holly Seale
**Additional Information:**	
**Question**	**Response**
**Financial Disclosure**<br><br>Enter a financial disclosure statement that describes the sources of funding for the	This research protocol was funded by the United States Centre for Disease Control and Prevention (CDC) through the cooperative agreement grant number 5U01GH1207. The funders had no role in study design, data collection and analysis, decision to publish, or manuscript preparation.

Powered by Editorial Manager® and ProduXion Manager® from Aries Systems Corporation

work included in this submission. Review the submission guidelines for detailed requirements. View published research articles from *PLOS ONE* for specific examples.<br><br>This statement is required for submission and **will appear in the published article** if the submission is accepted. Please make sure it is accurate.<br><br>**Unfunded studies**<br>Enter: *The author(s) received no specific funding for this work.*<br><br>**Funded studies**<br>Enter a statement with the following details:<br>• Initials of the authors who received each award<br>• Grant numbers awarded to each author<br>• The full name of each funder<br>• URL of each funder website<br>• Did the sponsors or funders play any role in the study design, data collection and analysis, decision to publish, or preparation of the manuscript?<br>• **NO** - Include this sentence at the end of your statement: *The funders had no role in study design, data collection and analysis, decision to publish, or preparation of the manuscript.*<br>• **YES** - Specify the role(s) played.<br><br>* typeset	
**Competing Interests**<br><br>Use the instructions below to enter a competing interest statement for this submission. On behalf of all authors, disclose any competing interests that could be perceived to bias this work—acknowledging all financial support and any other relevant financial or non-financial competing interests.<br><br>This statement is required for submission and **will appear in the published article** if the submission is accepted. Please make sure it is accurate and that any funding sources listed in your Funding Information	All authors report no conflicts of interest relevant to this article.

later in the submission form are also declared in your Financial Disclosure statement.<br><br>View published research articles from *PLOS ONE* for specific examples.<br><br>**NO authors have competing interests**<br><br>Enter: *The authors have declared that no competing interests exist.*<br><br>**Authors with competing interests**<br><br>Enter competing interest details beginning with this statement:<br><br>*I have read the journal's policy and the authors of this manuscript have the following competing interests: [insert competing interests here]*<br><br>* typeset	
**Ethics Statement**<br><br>Enter an ethics statement for this submission. This statement is required if the study involved:<br><br>• Human participants<br>• Human specimens or tissue<br>• Vertebrate animals or cephalopods<br>• Vertebrate embryos or tissues<br>• Field research<br><br>Write "N/A" if the submission does not require an ethics statement.<br><br>General guidance is provided below. Consult the submission guidelines for detailed instructions. **Make sure that all information entered here is included in the Methods section of the manuscript.**	The authors obtained written informed consent from all the participants before the KIIs and FGDs. The study protocol was reviewed and approved by UNSW ethical review committee (HC180517) and icddr,b institutional review board (PR 16090).

**Format for specific study types**<br><br>**Human Subject Research (involving human participants and/or tissue)**<br>• Give the name of the institutional review board or ethics committee that approved the study<br>• Include the approval number and/or a statement indicating approval of this research<br>• Indicate the form of consent obtained (written/oral) or the reason that consent was not obtained (e.g. the data were analyzed anonymously)<br><br>**Animal Research (involving vertebrate animals, embryos or tissues)**<br>• Provide the name of the Institutional Animal Care and Use Committee (IACUC) or other relevant ethics board that reviewed the study protocol, and indicate whether they approved this research or granted a formal waiver of ethical approval<br>• Include an approval number if one was obtained<br>• If the study involved *non-human primates*, add *additional details* about animal welfare and steps taken to ameliorate suffering<br>• If anesthesia, euthanasia, or any kind of animal sacrifice is part of the study, include briefly which substances and/or methods were applied<br><br>**Field Research**<br><br>Include the following details if this study involves the collection of plant, animal, or other materials from a natural setting:<br>• Field permit number<br>• Name of the institution or relevant body that granted permission	
**Data Availability**<br><br>Authors are required to make all data underlying the findings described fully available, without restriction, and from the time of publication. PLOS allows rare exceptions to address legal and ethical concerns. See the PLOS Data Policy and FAQ for detailed information.	No - some restrictions will apply

A Data Availability Statement describing where the data can be found is required at submission. Your answers to this question constitute the Data Availability Statement and **will be published in the article**, if accepted.<br><br>**Important:** Stating 'data available on request from the author' is not sufficient. If your data are only available upon request, select 'No' for the first question and explain your exceptional situation in the text box.<br><br>Do the authors confirm that all data underlying the findings described in their manuscript are fully available without restriction?	
**Describe where the data may be found in full sentences. If you are copying our sample text, replace any instances of XXX with the appropriate details.**<br><br>• If the data are **held or will be held in a public repository**, include URLs, accession numbers or DOIs. If this information will only be available after acceptance, indicate this by ticking the box below. For example: *All XXX files are available from the XXX database (accession number(s) XXX, XXX.).*<br>• If the data are all contained **within the manuscript and/or Supporting Information files**, enter the following: *All relevant data are within the manuscript and its Supporting Information files.*<br>• If neither of these applies but you are able to provide **details of access elsewhere**, with or without limitations, please do so. For example:<br><br>*Data cannot be shared publicly because of [XXX]. Data are available from the XXX Institutional Data Access / Ethics Committee (contact via XXX) for researchers who meet the criteria for access to confidential data.*<br><br>*The data underlying the results presented in the study are available from (include the name of the third party*	Data are available from the data repository committee at icddr,b. The dataset underlying the findings described in the paper cannot be shared publicly due to ethical restrictions related to protecting study participants' privacy and icddr,b's data access policy (https://www.icddrb.org/policies). icddr,b has a data repository maintains by the research administration. A copy of the complete dataset (anonymized and decoded) of this study will remain at the data repository. Interested researchers may contact Ms. Armana Ahmed, head of research administration (aahmed@icddrb.org), for approval and data access.

and contact information or URL).<br>• This text is appropriate if the data are owned by a third party and authors do not have permission to share the data.<br><br>* typeset	
Additional data availability information:	

Manuscript Click here to access/download;Manuscript;Manuscript_PLOS.doc

Title: Exploring hospital readiness for implementing the National TB infection control guidelines in two public tertiary care hospitals, Bangladesh

Running Title: Hospital readiness for implementing TB infection prevention and control guidelines

M. Saiful Islam[1,2*], Sayeeda Tarannum[1], Sayera Banu[1], Kamal Ibne Amin Chowdhury[1], Arifa Nazneen[1], Abrar Ahmad Chughtai[2], Holly Seale[2]

[1] Program for Emerging Infections, Infectious Diseases Division, icddr,b, Dhaka, Bangladesh

[2]School of Population Health, Faculty of Medicine and Health, University of New South Wales, Sydney, Australia

Keywords: Pulmonary tuberculosis, patient management, nosocomial infection, Infection prevention and control, Bangladesh

*Corresponding author: Program for Emerging Infections, Infectious Diseases Division, icddr,b, Dhaka, Bangladesh. Orcid id: https://orcid.org/0000-0002-5690-5971. Email: saiful@icddrb.org

Abstract

In high tuberculosis (TB) burden countries, health settings, including non-designated TB hospitals, host many patients with pulmonary TB. Bangladesh's National TB Control Program (NTP) aims to strengthen TB infection prevention and control (IPC) in health settings. However, there has been a failure to date to assess the readiness of hospitals to comply with the recommendations. To address this gap, our study examined healthcare workers (HCW) knowledge and attitudes towards TB IPC guidelines and their perceptions regarding the

hospitals' readiness, Bangladesh. Between January to December 2019, we conducted 16 key-informant interviews and four focus group discussions with HCW from two public tertiary care hospitals. In addition, we undertook a review of 13 documents [i.e. hospital policy, annual report, staff list, published manuscript]. Our findings showed that HCW acknowledged the TB risk and were willing to implement the TB IPC measures but identified key barriers impacting implementation. Gaps were identified in: policy (no TB policy or guidelines in the hospital), health systems (lack of TB IPC program, training and education, absence of healthcare-associated TB infection surveillance, low priority of TB IPC, no TB IPC monitoring and feedback, high patient load and bed occupancy, and limited supply of IPC resources) and behavioural factors (risk perception, compliance, and self-stigma). The additional service-level gap was the lack of electronic medical record systems. These findings highlighted that while there is a demand amongst HCW to implement TB IPC measures, the public tertiary care hospitals have got key issues to address. Therefore, the NTP may consider these gaps, provide TB IPC guidelines to these hospitals, assist them in developing hospital-level IPC manual, provide training, and coordinate with the ministry of health to allocate separate budget, staffing, and IPC resources to implement the control measures successfully.

Introduction

Healthcare workers (HCW) and other hospital occupants are at increased risk of tuberculosis (TB) infection in high TB burden countries [1]. Prior studies have shown that pulmonary TB patients stay on average 5.5 days in public tertiary care hospitals for TB diagnosis and treatment, including treatment for comorbidities [2]. In addition, pulmonary TB patients in inpatients wards with limited or no IPC measures have been shown to increase HCW exposure to TB, resulting in 42% of latent TB infections among HCW [3].

TB infection prevention and control (IPC) is one of the World Health Organization’s (WHO) 12 collaborative activities to prevent TB [4]. In low-and middle-income countries (LMIC), TB IPC is considered a critical strategy for TB prevention and control in health settings [5]. International TB IPC guidelines that target health settings can be traced back to the 1980s, and the most recent TB IPC guidelines was updated and published in 2019 by the WHO [6, 7]. As part of the WHO’s TB control strategy, many LMIC have developed national TB IPC guidelines; however, the implementation of these guidelines remains inconsistent due to multifaced factors prevailing in diverse health settings [7]. Bangladesh developed its national TB IPC guidelines in 2011, which includes a three-level hierarchy of control measures: administrative, environmental, and personal respiratory protection [8]. In 2019, Bangladesh ranked seventh among the 30 high TB burden countries globally [9]. The estimated incidence rate of all forms of TB per 100,000 population was 221 [9].

Bangladesh’s National TB control program (NTP) is working to strengthen TB IPC in health settings. However, to date, there has been a failure to assess the readiness of hospitals to implement TB IPC. To support NTP, it is crucial to understand the local context and broader health system factors, including behavioural and organisational factors [10]. An assessment of

target facilities to understand the barriers, leadership presence, attitudes of frontline HCW, and local leaders in TB IPC programs can support the tailoring and implementing guidelines [11, 12]. We adopted the WHO IPC Assessment Framework (IPCAF) tool to conduct the hospital readiness assessments for TB IPC [13]. The assessment aimed to examine HCW' knowledge and attitudes towards TB IPC guidelines and the readiness of two public tertiary care hospitals in Bangladesh to implement the guidelines.

Materials and methods

Study sites and participants

Between January and December 2019, a qualitative study was undertaken involving two public tertiary care hospitals: Hospital A and Hospital B. Key characteristics of the hospitals have been presented in Table 1. The rationale for selecting these hospitals was that they admitted approximately 400 adult pulmonary TB patients annually. Staff members involved in TB patient management were recruited from: medicine wards, directly observed therapy short-course (DOTS) clinics, radiology department, and administrative units. To recruit participants for interviews and focus group discussions (FGD), the team made a list of HCW who were members of hospital committees, those who were deemed to be influential in decision-making, knowledgeable, and experienced in dealing with infectious disease patients, and those who could affect or influence other's risk perception and practices. The team then selected those with the highest duration working in tertiary care hospitals.

Data collection

In order to get a rich understanding of the existing IPC practices, hospital infrastructure, and resources available for TB IPC, as well as explore the organisational readiness for change, the study included a hospital documents review, key-informant interviews (KII), and focus group

discussions (FGDs). The use of multiple data sources allowed us to triangulate the data. The KII and FGD guidelines were developed based on the IPCAF tool published by WHO in 2018 to assist the implementation of the WHO Core Components of IPC programs in health settings [14, 15]. The use of this tool in the guidelines helped us to detect relevant gaps in hospital readiness. A field team of three social scientists trained in qualitative research was employed to collect the data. The team has several years of experience working in hospital IPC. Before data collection, the team received a week-long training on data collection tools, participant recruitment, interacting with participants, and training on how to be reflexive, reflective, and minimise subjectivity during interviews and FGDs.

<u>Key-informant interviews</u>

Through KII, the team explored (i) hospital infrastructure, context, and readiness, (ii) HCW risk perception, concerns, attitudes and anticipated barriers regarding the TB IPC implementation; and (ii) the role that HCW (and others) can play in overcoming the barriers. The interviews were conducted in HCW' office room. The average duration of interviews was 49 minutes, and the interviews were audio-recorded. However, two key-informants refused to be recorded in the interviews, and then detailed hand notes were taken.

<u>Focus group discussions</u>

The FGDs were conducted in a hospital conference/meeting room where six to 10 people participated in each. Two team members conducted the focus groups: one moderated the session, and the other took detailed notes. Like KII, FGDs were also audio recorded. We convened the FGDs to elicit HCW perspectives on more complex phenomena (e.g., power relation, hospital priority, social and cultural aspects of TB, and TB among healthcare workers). The conversational nature during the FGDs allowed participants to share their perspectives and listen

to and reflect on other participant perspectives. The sessions ranged from 1hour 30 minutes to 2 hours.

Document review

Historical documents were reviewed to understand hospital characteristics, building design, in-patient-ward layout and renovation, bed capacity, and bed-occupancy rate. The reviewed documents included national TB IPC policy, national and hospital level health bulletins, published and unpublished reports; scientific manuscripts focused on study hospitals; and hospital staff lists.

Data analysis

All the data were collected in Bengali. We performed a descriptive analysis of the document review data. The team transcribed the audio-recorded interviews and FGDs verbatim and typed them into word. The audio recordings were repeatedly listened to, compared with the transcriptions to check for accuracy. Initially, two authors reviewed the field notes, two KIIs, and one FGD and developed a code list. The coding system was based on a literature review, study objectives, themes, and sub-themes related to TB IPC measures and emerging themes[16]. The transcribed data and field notes were transferred into text organising software (NVivo) to facilitate coding and analysis. The lead author then read the interviews and FGDs and coded the excerpts of texts according to the code list. When new information could not be coded with the existing code list, the author added new codes, and the process continued until the end. The intercoder reliability was achieved through discussions among the two coders about code definitions and emerging themes[17]. The coded data were then categorised under different themes that emerged, and according to each theme, a summary was developed in English[16]. All the authors reviewed the themes and sub-themes for consensus and reliability. Our analytical

framework included three categories consisting of 10 dimensions- (a) policy level factors: i) TB IPC guidelines; (b) health system factors: ii) TB IPC programs; iii) education and training; iv) HAI surveillance; v) Multimodal strategies including system and culture change; vi) monitoring and evaluation; vii) workload, staffing, and bed occupancy; viii) the built environment, materials and equipment, and (c) behavioural level factors: ix) Risk perception; x) compliance with personal protective equipment; and xi) Stigma.

Study definitions

Adopting from Weiner, we defined organisational readiness as the extent to which organisations are prepared infrastructurally, administratively, and with resources, and HCW are prepared psychologically, behaviorally and willing to accept the changes due to TB IPC implementation [18].

Ethical approval

The authors obtained written informed consent from all the participants before the KIIs and FGDs. The study protocol was reviewed and approved by UNSW ethical review committee (HC180517) and icddr,b institutional review board (PR 16090).

Results

The team conducted 16 KIIs and four FGDs, where 51 people participated (16 HCW as key informants and 35 as FGD participants). Among all participants, 29 were doctors, 20 were nurses, and two were ancillary workers. The participants' mean age was 39 years (standard deviation (SD)-9.6), and 32 were male. The key-informants had an average of 20.6 years (SD:10.8) of work experience, and the FGD participants had an average of 6.8 years (SD:6.3) of work experience in public tertiary care hospitals. We report policy level, hospital level, and behavioural level factors that may impact the implementation of TB IPC measures.

Policy level factors

TB IPC guidelines

No TB IPC guidelines were available in the study hospitals. Almost all the key informants and FGD participants said they were unaware of national TB IPC guidelines. One of the key informants mentioned that he had been working in the facility since 1957 and has never seen a copy of the TB IPC guidelines. The participants added that they did know about the standard IPC measures.

Hospital level factors

TB IPC programs

All the participants said that the study hospitals lacked a TB IPC program with a dedicated IPC lead, focal person, committee, or coordination body. The participants reportedly did not receive any demonstrable support from NTP for TB IPC activities. One of the participants from an FGD said,

> *To my knowledge, there is no TB IPC committee in this hospital. There might be a committee on pen and paper, but I did not hear about this or did not see the committee's*

activity. There was no publicity about any TB IPC committee in the hospital. If there was a TB IPC committee, I must be involved there.

There was no separate budget for the TB IPC program in the hospital. FGD participants mentioned that they received funding according to the number of beds in the hospital, not according to the number and types of patients they admit and treat.

TB IPC Education and Training

None of the study participants reported that they received any dedicated training for TB IPC. One key informant reasoned that since the study hospitals were not TB specialised hospitals and TB IPC was not a priority, they did not receive training. Some doctors mentioned that they received education and training on TB patient treatment and management, and there were brief discussions on TB IPC. One FGD participant mentioned that all levels of HCW did not have access to any training. Only a few people, particularly the doctors, received training repeatedly. The nurses mentioned that they often provide health education, including cough etiquette and respiratory hygiene, to patients and family caregivers, but no TB IPC training was offered to patients.

The doctors in FGD highlighted the necessity of TB IPC training as-

> *We need training on TB IPC. Since the TB IPC strategies may update over time, we need refresher training every year. The authority may train a few senior HCW on TB IPC at the national level. These trained HCW can train others in the hospital at the local level. We need to follow the training for trainers (ToT) approach. Following this approach, the trainers will learn more and be efficient when providing training to other HCW.*

Healthcare-associated TB infection surveillance

All the participants mentioned that there was no surveillance for healthcare-associated TB infection in the hospitals. They mentioned that surveillance would require financial and human resources, and the hospital lacked TB-specific resources. Moreover, they added that they never received any instruction from the health ministry to run surveillance on healthcare-associated TB infection.

Multimodal strategies including system and culture change

The key informants mentioned that public tertiary care general hospital served a variety of patients. Therefore, these hospitals have diverse priorities. Two of the key informants said that the primary focus of the hospitals was patient investigation and treatment. Therefore, TB IPC remained a neglected part. One of the KII participants who was the head of medicine wards mentioned-

> *This is a public tertiary care teaching hospital. We do not only treat TB patients, but we also admit all kinds of patients. It is not possible to put so much effort into TB patients. TB screening and isolation on admission is possible only in TB specialty hospitals, not here (tertiary care general hospital). We receive 1 percent or 1.5 percent of the total patients with TB. We will not be able to implement TB IPC recommendations for this small proportion of TB cases. It (TB IPC) is not possible to implement here.*

The nurses from one FGD informed us that they do not have easy access to discuss IPC with the hospital director. One of the FGD participants shared about the chain of information sharing as-

> *(For any infection control issue) We need to talk to the assistant registers at the inpatient wards first, and then the assistant registers talk to the registers. The registers then talk to the professors, and the professors then discuss the issue with the hospital director.*

Monitoring and feedback

The participants informed us that the hospitals lacked a well-defined and targeted TB IPC monitoring and feedback plan. The was no evaluation of patient and HCW safety culture in the hospitals. One of the key informants mentioned that there might be a monitoring and feedback culture for clinical practices, but none exists for TB IPC. One participant mentioned that

> *As there was no implementation of TB IPC in the hospital, there was no monitoring and feedback systems observed.*

Workload, staffing, and bed occupancy

The study participants mentioned that they had insufficient human resources and, therefore, they were overburdened and had to serve a high number of patients every day. The document review showed that the hospitals had a bed occupancy rate of more than 150 annually (Table 1). The number of patients reportedly always exceeded the number of beds in inpatient wards and occasionally increases up to 10 folds on admission days. One of the key informants mentioned that he had been using the space of 12 patients for 65 patients during the study period. Due to the excessive number of patients in a limited space, it was reportedly impossible to maintain a minimum spacing of one meter between patients.

> *Due to the high patient load, we cannot follow standard IPC while dealing with pulmonary TB patients. Pulmonary TB patients stay with patients without TB in the inpatient wards. In most cases, we refer TB patients to TB specialty hospitals. However, the TB specialised hospitals often send them back to our hospital, or the patients themselves come back to our hospital and make the ward crowd.*

Built environment, materials, and equipment for TB IPC

All the participants mentioned that the inpatient wards had been spacious with large doors and windows; however, the renovation of the old buildings, the establishment of new buildings, and high patient load affected the open space, inpatient wards layout, cross ventilation, and access to sunlight. The participants added that they could not follow the standard IPC practice while dealing with infectious patients due to limited space and supply of IPC resources. One of the participants who was in a senior position at the hospital mentioned-

> *We need facilities and resources for TB IPC implementation. We would require a separate space to receive presumptive TB patients, separate room to isolate them so that other general patients cannot be exposed to the presumptive TB patients.*

Both the hospitals were naturally ventilated. The nurses informed us that they often had to close doors and windows to avoid visitors and family caregivers in the ward. Moreover, they often kept the doors and windows closed to allow patients on the floor near the doors and windows. To avoid direct sunlight, some patients often preferred covering the windows with a curtain that hindered airflow. In the evening, the nurses reportedly closed the windows to prevent mosquitoes. They also shared that the patients or the ancillary workers often closed the doors and windows during the winter months to avoid cold winter air.

The study participants informed us that there was no supply of masks for patients in the hospital. HCW often recommended the patient with pulmonary TB should buy a mask. The participants informed us that while there was a supply of medical masks for HCW, staff had to raise a requisition for masks to the hospital store. There was no supply of N95 respirators.

The key informants also informed us that the hospitals lacked an electronic medical record (EMR) system, which delays TB/patient management. The participants mentioned that the documentation of patients' information is done manually using pen and paper, leading to a long queue of patients in the outpatient department and diagnostic facilities. One of the key informants described the scenario as-

> *Every morning, there was a long queue of patients to buy consultation tickets near the outpatient department. Once the patient visits the doctor and receives a prescription for any pathological or X-ray tests, the patient needs to wait in another long queue in the diagnostic facilities. Due to the long waiting time in multiple queues, some patients cannot complete all the tests on the same day. If we could digitalise the system, we could minimise patients hang around time and contamination of hospital areas with infectious droplets and airborne bacteria.*

Healthcare workers-level factors

Perception of nosocomial TB

All the participants from KIIs and FGDs said they were at increased risk of TB infection due to prolonged and continued exposures in inpatient wards. The participants argued that HCW visit TB patients almost every day for 365 days a year. Most of the participants provided examples of TB disease among HCW, including multi-drug resistant TB. One of the key informants from a medicine ward mentioned that nurses were at the highest risk of TB infection due to their direct and long exposures to TB patients. Similar findings were also reflected in an FGD with nurses. One of the FGD participants said,

> *We are at 100% risk of TB infection. There is no TB screening for presumptive TB patients in this hospital. There are always 1 to 4 TB patients in the ward. We do not take precautions,*

such as wearing a mask when dealing with presumptive TB patients. Therefore, we are always at risk of TB infection.

The HCW from the pediatric wards mentioned that they were at lower risk of nosocomial TB. They argued that pediatric patients could not produce cough and therefore were less likely to contaminate the air when compared with adult pulmonary patients. Therefore, they were not concerned about TB infection and did not take any precautions to prevent TB transmission. They reportedly visited presumptive TB patients without masks or respirators.

Social and cultural aspects of TB and stigma

TB was still considered a stigmatised disease. The participants mentioned that HCW with TB disease faced discrimination in the family, society, and workspace. To avoid stigma and discrimination, HCW often hide their TB disease. One of the key informants (nursing-in-charge) highlighted how and why HCW with TB often hide their TB status-

> *Doctors, nurses, and allied HCW are getting infected with TB. They often hide that they have TB. To my knowledge, there are five nurses with TB disease in the hospital. They requested me not to tell their TB disease to others. They are unmarried girls. People do not want to marry girls with TB disease; even they become disease-free after treatment. Their family members also avoid them. Their colleagues also avoid them in the workspace. To avoid discrimination and stigma, HCW often hide their TB disease*.

The reported role that healthcare workers can play in implementing TB IPC

The participants pointed out several factors that can be followed to better implement the TB IPC guidelines (Table 2). The nurses said that the doctors take a patient history and may be aware of presumptive TB patients. The doctors may inform the nurses about presumptive TB patients, and the nurses can take necessary TB IPC measures. The nurses also mentioned that they could put a

sign near a TB patient bed so that HCW can take standard precautions while visiting a TB patient. The nurses also added that they could use curtains around presumptive TB patients' beds as they have space limitations for isolation. One of the key-informant said-

> *The patients enter the hospital through outdoors and emergency departments. We want TB IPC implementation, including cough screening at these two places. We also need to introduce the TB IPC control measures among the senior and junior level HCW. There should be frequent training so that HCW can attain the training by rotation. We also need infrastructure for TB screening and isolation. There is a need to collaborate with the government, hospital, and private sectors to reduce TB infection in the hospital.*

Lastly, it was suggested that local hospital HCW should be invited to the national TB IPC guidelines development committee. He said,

> *Because HCW work at the local level and implement guidelines, their opinions should be reflected in guidelines. The central policymakers develop guidelines based on their perceptions and theoretical knowledge. Due to the lack of local level HCW views in the policy, the hospital contexts may not be reflected in the policy. Therefore, the policy may fail during the implementation stage.*

Discussion

This study identified gaps in the TB IPC policies, hospital infrastructure, IPC supplies and equipment, which impact the TB IPC implementation. The findings showed both the hospitals lacked TB IPC and surveillance programs, which is consistent with a nationally representative survey that found facilities lacked 85% of standard precaution components required for TB management and IPC in Bangladesh [19]. In the study facilities, identifying presumptive TB patients on admission was hindered by a lack of cough screening and a case definition. Separation and isolation of presumptive TB patients were affected by limited space, high patient load, limited supply of consumable IPC resources such as masks and respirators, HCW' IPC training gaps, and absence of isolation guidelines. As outlined in the WHO's guidelines on Core Components of IPC, national IPC programs must ensure that infrastructure and IPC resources are available to implement IPC measures successfully [15]. The challenge is translating these recommendations to hospitals that have high bed-occupancy rate of more than 150%, overcrowding on admission days, and understaffing. Since these hospitals receive funding according to the bed capacity, it is likely that they continue to exhaust their resources and so cannot meet the TB IPC needs of staff when caring for the extra patients. This situation has probably been exacerbated due to the COVID-19 pandemic, with resources being diverted towards pandemic management [20, 21].

Participation in hospital infection surveillance systems has previously been linked to reductions in healthcare-associated TB infections [22]. While both of the study hospitals have laboratory capacities to support surveillance, it is not occuring. While this was beyond the scope of the project to explore the perspectives of those in government, it may be speculated that awareness about the burden of nosocomial TB in tertiary care hospitals may be low, leading to a failure to

prioritize the issue. If surveillance data was available it could be shared with NTP, hospital and department managers to motivate them to allocate resources for TB-specific IPC programs.

It is well documented that diagnostic delays increase the length of hospital stay for pulmonary TB patients [23]. From our previous work, we identitified that it took around five days to diagnose a patient with TB from admission, while the median length of hospital stay for a pulmonary TB patient without TB treatment was 5.4 days [2]. In the two study hospitals, we identified several key factors that are leading to the delay in diagnosing TB including: (1) a high number of patients; (2) patient admission after office hours; (3) opening time of diagnostic facilities (8:30 AM to 3 PM), and (4) the lack of an EMR system. In alignment with previous calls for action, we feel that it is critical that action be taken to reduce the time taken to diagnose untreated pulmonary TB patients [23]. As recommended by the participants, an EMR system could help to minimise the time these patients spend at the patient registration desk and at diagnostic facilities, as well as helping to improve reporting times. The implementation of EMR systems is aligned with the Bangladesh government's national policy [24].

The WHO Core Components of IPC guidelines recommends that HCWs should receive education and training on IPC guidelines recommendations[6]. Amongst our participants, none reportedly received any education and training on TB IPC. The lack of training among nurses and ancillary workers was perceived as barriers to TB IPC; several participants called for the TB IPC guidelines and training on recommendations. Organising training at these hospitals, promoting core and TB IPC and engaging all HCW, including doctors, nurses, and ancillary workers, may build partnership in the IPC program to help to implement the TB IPC measures. Beyond training, it is also important to support communication within the hospital. Our participants indicated that there was a communication gap between doctors, nurses, and other

ancillary workers. This may be contributing to low uptake of IPC practices, particularly around the separation and isolation of presumptive TB patients. Communication between hospital leadership, doctors, nurses, and other ancillary workers can support adherence to IPC measures [25]. Prior studies have shown that receiving instructional feedback from senior colleagues can motivate other staff members to implement IPC measures [26].

Risk perceptions towards TB disease can also have an impact on acceptance and use of TB IPC measures [27]. Whilst our participants were aware that they are being exposed to pulmonary TB patients and that they were at risk of TB infection; their risk perception did not translate into action. Limited understanding about disease transmission and poor risk perception may result in non-compliance, poor health-seeking behaviour, and treatment that may result in TB transmission in the hospital [28]. The perception amongst participatnts that health workers could be a source of TB transmission in the hospital was poor, with many HCW continuing to work when they were sick or hide their TB disease status while working in the hospital. The reasons for continuing working while they were still sick could be their fear of losing salary when they will be on sick leave; and fear of being re-assigned or even fired as noted by Delft et al[29]. The reason to hide TB status could be due to the fear of being stigmatized by co-workers. In a study among 879 HCW in South Africa, 19% of the respodents agreed that HCWs with presumptive TB are stigmatized in hospitals[30]. The HCW with TB disease can be a source of infection in the inpatient ward and can affect the effectiveness of TB IPC measures if implemented.

This study has limitations. We conducted this study in only two hospitals, and therefore, the study findings are very context specific. The findings presented study can be an indicative of hospital readiness for TB IPC implementation in similar hospitals in Bangladesh, and other low-

income high TB burden countries. Moreover, the study findings are consistent with a prior study that assessed the TB IPC in 11 TB specialized hospitals, tertiary care hospitals, and TB clinics [31]. Secondly, we did not interview any stakeholders from the ministry of health or NTP. Therefore, we could not understand the national-level factors that may influence TB IPC implementation in the local level tertiary care hospitals. Finally, this assessment identified strengths and weaknesses in health systems and services to adopt TB IPC, and the context may change over time and impact effectiveness.

In conclusion, this study found that while there was a positive attitude towards TB IPC and staff members are willing to implement strategies, there are key barriers that need to be addressed. Currently the study hospitals will struggle to implement the TB IPC measure due to high patient load, limited space, and lack of TB IPC specific budget and resources. Establishing new hospitals to reduce patient load is time and resource expensive. Therefore, we recommend leveraging existing resources for the implementation of TB IPC measures. We recommend that the space limitation for presumptive TB patient isolation be minimised by setting up an isolation area in the veranda or corridors with a large open area facing outside. If it is not possible during the rainy monsoon season, a curtain can separate patients inside the inpatient ward as recommended by the study participants. All coughing patients should wear a mask until they confirm the diagnosis. Besides, the ancillary workers can be engaged in IPC programs to keep the doors and windows open to ensure natural ventilation. The national TB program or other national and international organisations working on TB elimination can visit these hospitals, form TB IPC committees following the recommendations made by the participants and organise TB IPC training in the hospitals so that the HCW fully understand TB IPC guidelines recommendations and build the task into their daily activities. Some nurses or senior nursing

students can be trained on TB advocacy and counselling to provide health education, including respiratory hygiene and counselling, to support TB patients and their family caregivers. All HCW should be given information and motivation, including a care package, so they feel encouraged to undergo TB diagnostic investigation if they have signs and symptoms suggestive of TB. Finally, we recommend public tertiary care hospitals receive a budget according to the annual number of patients, not according to the allocated beds for the hospitals.

Acknowledgments

We want to thank the study hospitals' directors and all the study participants for their time and respect. icddr,b acknowledges with gratitude the commitment of CDC to its research efforts. icddr,b is also grateful to the Governments of Bangladesh, Canada, Sweden, and the UK for providing core/unrestricted support.

Financial support

This research protocol was funded by the United States Centre for Disease Control and Prevention (CDC) through the cooperative agreement grant number 5U01GH1207. The funders had no role in study design, data collection and analysis, decision to publish, or manuscript preparation.

Conflicts of interest

All authors report no conflicts of interest relevant to this article.

References

1. World Health Organization. Global Tuberculosis Report. Geneva: World Health Organization, 2019.
2. Islam MS, Banu S, Tarannum S, Chowdhury KIA, Nazneen A, Islam MT, et al. Examining patient management and healthcare workers exposures to pulmonary TB patients in two public tertiary care hospitals, Bangladesh. PloS one. 2021;Submitted.
3. Islam MS, Chughtai AA, Nazneen A, Chowdhury KIA, Islam MT, Tarannum S, et al. A tuberculin skin test survey among healthcare workers in two public tertiary care hospitals in Bangladesh. PloS one. 2020;15(12):e0243951. Epub 2020/12/18. doi: 10.1371/journal.pone.0243951. PubMed PMID: 33332458; PubMed Central PMCID: PMCPMC7745963.
4. World Health Organization. Implementing the WHO Policy on TB Infection Control in Health-Care Facilities, Congregate Settings and Households A framework to plan, implement and scale-up TB infection control activities at country, facility and community level WHO Policy on TB Infection Control in Health-Care Facilities, Congregate Settings and. Geneva: World Health Organization, The tuberculosis coalition for technical asistance, Centers for disease control and prevention, United States Agency International Development, 2010.
5. Reuben Granich, Nancy J. Binkin, William R. Jarvis, Patricia M. Simone, Hans L Rieder, Marcos A Espinal, et al. Guidelines for the prevention of Tuberculosis in healthcare facilities in resource-limited settings. Geneva, Switzerland: World Health Organization; 1999.
6. World Health Organization. WHO guidelines on tuberculosis infection prevention and control, 2019 update. Geneva, World Health Organization: World Health Organization, 2019 Contract No.: WHO/CDS/TB/2019.1.
7. Islam MS, Chughtai AA, Banu S, Seale H. Context matters: An analysis of the implementation of tuberculosis infection prevention and control guidelines in health settings in seven high TB burden countries. Journal of Infection and Public Health. 2021;(In Submission).
8. National Tuberculosis Control Programme. National Guidelines for Tuberculosis Infection Control. Dhaka, Bangladesh: National Tuberculosis Control Programme, USAID. TB CARE II, Bangladesh, World Health Organization, 2011.
9. World Health Organization. Global Tuberculosis Report. Geneva: World Health Organization, 2020.
10. Petticrew M. When are complex interventions 'complex'? When are simple interventions 'simple'? European journal of public health. 2011;21(4):397-8. Epub 2011/07/21. doi: 10.1093/eurpub/ckr084. PubMed PMID: 21771736.
11. Baker R, Camosso-Stefinovic J, Gillies C, Shaw EJ, Cheater F, Flottorp S, et al. Tailored interventions to overcome identified barriers to change: effects on professional practice and health care outcomes. The Cochrane database of systematic reviews. 2010;(3):Cd005470. Epub 2010/03/20. doi: 10.1002/14651858.CD005470.pub2. PubMed PMID: 20238340; PubMed Central PMCID: PMCPMC4164371.
12. Flodgren G, Parmelli E, Doumit G, Gattellari M, O'Brien MA, Grimshaw J, et al. Local opinion leaders: effects on professional practice and health care outcomes. The Cochrane database of systematic reviews. 2011;(8):Cd000125. Epub 2011/08/13. doi: 10.1002/14651858.CD000125.pub4. PubMed PMID: 21833939; PubMed Central PMCID: PMCPMC4172331.
13. Storr J, Twyman A, Zingg W, Damani N, Kilpatrick C, Reilly J, et al. Core components for effective infection prevention and control programmes: new WHO evidence-based recommendations. Antimicrobial Resistance & Infection Control. 2017;6(1):1-18.

14. World Health Organization. Improving infection prevention and control at the health facility: interim practical manual supporting implementation of the WHO guidelines on core components of infection prevention and control programmes. Geneva: World Health Organization, 2018.
15. World Health Organization. Guidelines on Core Components of Infection Prevention and Control Programmes at the National and Acute Health Care Facility Level. Geneva: World Health Organization., 2016.
16. Braun V, Clarke V. Using thematic analysis in psychology. Qualitative Research in Psychology. 2006;3(2):77-101. doi: 10.1191/1478088706qp063oa.
17. O'Connor C, Joffe H. Intercoder Reliability in Qualitative Research: Debates and Practical Guidelines. International Journal of Qualitative Methods. 2020;19:1609406919899220. doi: 10.1177/1609406919899220.
18. Weiner BJ. A theory of organizational readiness for change. Implementation science : IS. 2009;4:67. Epub 2009/10/21. doi: 10.1186/1748-5908-4-67. PubMed PMID: 19840381; PubMed Central PMCID: PMCPMC2770024.
19. Biswas MAAJ, Hassan MZ, Monjur MR, Islam MS, Chowdhury F, Banu S, et al. Assessment of pre-COVID-19 standard precaution related to infection prevention readiness of health facilities in Bangladesh: Findings from a national cross-sectional survey. Unpublished manuscript. 2021.
20. Alene KA, Wangdi K, Clements ACA. Impact of the COVID-19 Pandemic on Tuberculosis Control: An Overview. Tropical medicine and infectious disease. 2020;5(3). Epub 2020/07/30. doi: 10.3390/tropicalmed5030123. PubMed PMID: 32722014; PubMed Central PMCID: PMCPMC7558533.
21. Burki T. Global shortage of personal protective equipment. The Lancet Infectious diseases. 2020;20(7):785-6. doi: 10.1016/S1473-3099(20)30501-6. PubMed PMID: 32592673.
22. Zingg W, Holmes A, Dettenkofer M, Goetting T, Secci F, Clack L, et al. Hospital organisation, management, and structure for prevention of health-care-associated infection: a systematic review and expert consensus. The Lancet Infectious Diseases. 2015;15(2):212-24. doi: https://doi.org/10.1016/S1473-3099(14)70854-0.
23. Zetola NM, Macesic N, Modongo C, Shin S, Ncube R, Collman RG. Longer hospital stay is associated with higher rates of tuberculosis-related morbidity and mortality within 12 months after discharge in a referral hospital in Sub-Saharan Africa. BMC Infectious Diseases. 2014;14(1):409. doi: 10.1186/1471-2334-14-409.
24. Ahmed MM. OP-ED: The urgent need for electronic medical records. The Daily Star. 2020 Sept 12;Sect. Op-Ed.
25. Sinuff T, Cook D, Giacomini M, Heyland D, Dodek P. Facilitating clinician adherence to guidelines in the intensive care unit: A multicenter, qualitative study. Critical care medicine. 2007;35(9):2083-9. Epub 2007/09/15. doi: 10.1097/01.ccm.0000281446.15342.74. PubMed PMID: 17855822.
26. Turnberg W, Daniell W, Simpson T, Van Buren J, Seixas N, Lipkin E, et al. Personal healthcare worker (HCW) and work-site characteristics that affect HCWs' use of respiratory-infection control measures in ambulatory healthcare settings. Infect Control Hosp Epidemiol. 2009;30(1):47-52. Epub 2008/12/03. doi: 10.1086/592707. PubMed PMID: 19046059.
27. Woith W, Volchenkov G, Larson J. Barriers and motivators affecting tuberculosis infection control practices of Russian health care workers. The international journal of tuberculosis and lung disease : the official journal of the International Union against Tuberculosis and Lung Disease. 2012;16(8):1092-6. Epub 2012/06/06. doi: 10.5588/ijtld.10.0779. PubMed PMID: 22687261.
28. Maroldi MAC, Felix AMdS, Dias AAL, Kawagoe JY, Padoveze MC, Ferreira SA, et al. Adherence to precautions for preventing the transmission of microorganisms in primary health care: a qualitative study. BMC Nurs. 2017;16:49-. doi: 10.1186/s12912-017-0245-z. PubMed PMID: 28919838.

29. von Delft A, Dramowski A, Khosa C, Kotze K, Lederer P, Mosidi T, et al. Why healthcare workers are sick of TB. Int J Infect Dis. 2015;32:147-51. Epub 2015/03/27. doi: 10.1016/j.ijid.2014.12.003. PubMed PMID: 25809771.
30. Engelbrecht M, Rau A, Kigozi G, Janse van Rensburg A, Wouters E, Sommerland N, et al. Waiting to inhale: factors associated with healthcare workers' fears of occupationally-acquired tuberculosis (TB). BMC Infectious Diseases. 2019;19(1):475. doi: 10.1186/s12879-019-4115-z.
31. Nazneen A, Tarannum S, Chowdhury KIA, Islam MT, Islam SMH, Ahmed S, et al. Implementation status of national tuberculosis infection control guidelines in Bangladeshi hospitals. PloS one. 2021;16(2):e0246923. doi: 10.1371/journal.pone.0246923.

Table 1: Key characteristics of the study hospitals, 2019

Characteristics	Hospital A	Hospital B
Location	• Located in Barisal.<br>• 123 kilometres away from Dhaka, the capital city of Bangladesh	• Located in Rajshahi.<br>• which is 198 kilometres away from Dhaka
Number of beds	• 1000 bed-hospital	• 1200-bed hospital
Number of patients (approximate) receive treatment	• 406,400 as outpatients,<br>• 117,263 as emergency patients, and<br>• 121,891 as in-patient	• 806,541 as outpatients,<br>• 12,154 as emergency patients, and<br>• 175,868 as inpatients
Number of Doctors	• 323	• 395
Number of Nurses	• 768	• 1145
Number of ancillary care workers	• 392	• 514
Bed occupancy rate	• In 2018, the bed-occupancy rate was 155.8%	• The bed occupancy rate in hospital B was 151.5%.
DOTS clinic	• Present within the hospital premises<br>• Provide diagnosis and treatment support to TB patients between 8:30 to 2 PM during the weekdays	• Present within the hospital premises<br>• Provide diagnosis and treatment support to TB patients between 8:30 to 2 PM during the weekdays
X-ray	• Inside the hospital facilities that runs 24-hours a day	• Inside the hospital facilities
GeneXpert	• GeneXpert facilities available in the neighbouring TB specialty hospitals	• GeneXpert facilities available in the neighbouring TB specialty hospitals
Surveillance for healthcare-associated TB infection	• Not present	• Not present
TB IPC committee	• Not present	• Not present
TB IPC guidelines	• Not present	• Not present
General IPC guidelines	• Not present	• Not present
TB IPC training	• Not present	• Not present
Cough screening	• Not present	• Not present
Separation of patients with pulmonary TB	• Partially present<br>• TB patients with known pulmonary TB	• Partially present<br>• TB patients with known pulmonary TB occasionally

	occasionally separated in the corner of the inpatient ward	separated in the corner of the inpatient ward
Isolation	• Piloted an isolation ward for TB for a year. Not available now.	• Piloted an isolation ward for TB for a year. Not available now.
Ventilation	• Natural ventilation<br>• Ceiling fans available	• Natural ventilation<br>• Ceiling fans available
Visitor control	• There is a policy on visiting hours.<br>• No implementation of visitor control	• There is a policy on visiting hours.<br>• No implementation of visitor control
Supply of PPE	• A limited supply of IPC resources<br>• No TB specific IPC supplies<br>• No supply of N95 resources	• A limited supply of IPC resources<br>• No TB specific IPC supplies<br>• No supply of N95 resources

Table 2: Participants' recommendations for TB IPC implementation in health settings.

TB IPC measures	Recommendations
**Administrative**	
Guideline development	• Engage frontline caregivers in national TB IPC guideline development.
TB IPC committee	• Form a committee by involving HCW from different professions and levels to facilitate and accelerate IPC activities in the hospital.<br>• The hospital director, nursing superintendent, and ward-in-charge could be added to the hospital TB IPC committee.<br>• Infection control coordination body
Cough screening	• Set up screening at the outdoors and emergency departments.<br>• There should be a screening system before admitting a TB patient to the inpatient ward.
Separation	• In the inpatient ward, allocate two beds for presumptive TB patients where sufficient cross ventilation can be ensured.<br>• Open a unit or ward for TB patients in tertiary care hospitals.<br>• Establish a waiting area near the outdoors.
Isolation	• Based on symptom screening, presumptive TB patients can be sent to an isolation ward.
Crowd control	• Control visitors<br>• Allow patients according to bed numbers.<br>• Initiate identity cards for visitors.

	• Only one adult visitor per patient can be allowed.
Training	• Training for trainers<br>• Hospital-based training.<br>• Ensure participation from all wards.<br>• Annual refresher training<br>• Using audio-visuals, educate patients and family caregivers about transmission pathways of TB, cough hygiene.<br>• A monthly discussion on IPC among HCW
Infrastructure	• TB specialty hospitals should be enriched so that they can treat TB patients with comorbidities.
**Environmental**	
Ventilation	• Ensure opening the doors and windows.<br>• Improve inpatient ward layout to ensure proper ventilation.
**Respiratory protection**	• Mask can be provided to presumptive TB patients on admission.
